# Clinical decision rules in primary care: necessary investments for sustainable healthcare

**DOI:** 10.1017/S146342362300021X

**Published:** 2023-05-02

**Authors:** Jorn S. Heerink, Ruud Oudega, Rogier Hopstaken, Hendrik Koffijberg, Ron Kusters

**Affiliations:** 1 Department of Clinical Chemistry and Haematology, Jeroen Bosch Hospital, ‘s-Hertogenbosch, the Netherlands; 2 Department of Health Technology and Services Research, Technical Medical Centre, University of Twente, Enschede, the Netherlands; 3 Julius Centre for Health Sciences and General Practice, University Medical Centre Utrecht, the Netherlands; 4 Star-shl Diagnostic Centres, Etten-Leur, the Netherlands

**Keywords:** clinical decision rule, D-dimer, deep vein thrombosis, Oudega rule, point-of-care, wells rule

## Abstract

Clinical judgement in primary care is more often decisive than in the hospital. Clinical decision rules (CDRs) can help general practitioners facilitating the work-through of differentials that follows an initial suspicion, resulting in a concrete ‘course of action’: a ‘rule-out’ without further testing, a need for further testing, or a specific treatment. However, in daily primary care, the use of CDRs is limited to only a few isolated rules. In this paper, we aimed to provide insight into the laborious path required to implement a viable CDR. At the same time, we noted that the limited use of CDRs in primary care cannot be explained by implementation barriers alone. Through the case study of the Oudega rule for the exclusion of deep vein thrombosis, we concluded that primary care CDRs come out best if they are tailor-made, taking into consideration the specific context of primary health care. Current CDRs should be evaluated frequently, and future decision rules should anticipate the latest developments such as the use of point-of-care (POC) tests. Hence, such new powerful diagnostic CDRs could improve and expand the possibilities for patient-oriented primary care.

## Background

In a typical consultation, general practitioners (GPs) address three problems and make eight decisions (Ebell, 2010). To facilitate this process, they often use many mental shortcuts (heuristics) before coming to a diagnosis, in addition to history taking and physical examination (Phillips, 2010b). In this way, many accurate diagnoses are made, but occasionally mistakes lie in wait. Especially if symptoms are vague or atypical, there is a risk of taking a wrong diagnostic turn. In such cases, relying on clinical examination alone does not provide an adequate rationale for clinical decision-making.

Here, clinical decision rules (CDRs) can provide relief. These rules are developed for physicians to objectivate and guide their decision-making by facilitating the refinement process of the work-through of differentials that follows an initial suspicion (Phillips, 2010b). In relevant cases, they serve as evidence-based practical guidelines to make clinical practice more tangible (Green, 2013; Phillips, 2010a). The application of decision rules improves the clinician’s ability to treat patients consistently and to focus on the patient’s clinical context (Gaddis *et al.,* 2007). Additionally, the use of CDRs can avoid diagnostic errors and prevents unnecessary tests (Phillips, 2010b; Reed, 2006).

The term ‘Clinical Decision Rule’ has many definitions, but fundamentally it is a tool that quantifies and combines simple available clinical indicators in order to define a new parameter. In primary care, this parameter can be generated by combining patient signs, symptoms, and additional readily available laboratory, function, or imaging test results and can be used to estimate a score related to the probability of the presence or absence of a specific disease (diagnosis) or outcome (prognosis). Subsequently, a concise diagnostic or therapeutic ‘course of action’ or a preventive strategy should be undertaken (le Gal *et al.,* 2012). In this way, clinical behaviour is promoted leading to a more structured and consistent decision-making, improving quality of care and patient satisfaction while reducing unnecessary costs, preferably combined with a reduction of practice variation among doctors (Reilly and Evans, 2006). The use of decision rules can help junior physicians to develop their clinical judgement but can also assist more experienced doctors in rapidly making decisions on complex diagnoses or potential life-threatening conditions (Carmelli *et al.,* 2018). In the end, however, the main value of a CDR lies in reducing the complexity of combining symptoms and signs to decision recommendations in terms of, typically, a ‘rule-out’ without further testing, a need for further testing, or a specific treatment (Ebell, 2010).

Ideally, CDRs are perfectly suited to be implemented in a primary care setting. The mere opportunity to use a CDR helps GPs find their way in the wide variety of patients presenting to them that suffer from an even wider variety of often early symptoms (le Gal *et al.,* 2012) for which a wide range of diagnostic and treatment options is available. As a gatekeeper, one of the main strengths of GPs is avoiding unnecessary referrals for comprehensive diagnostics and specialist consultations, and CDRs are particularly suitable for this purpose (Reed, 2006). Nonetheless, even though numerous CDRs were developed over the past few decades, their use in daily primary care is limited to only a few isolated rules.

In the present work, we focussed on providing insight into the challenges surrounding the implementation of CDRs as well as challenges in everyday use of well-established CDRs, using the CDR for deep vein thrombosis (DVT) in primary care patients as a case study. Lastly, we used these insights to formulate suggestions for a more effective application of CDRs in primary care.

## From derivation to implementation of a viable CDR

**Figure 1.**
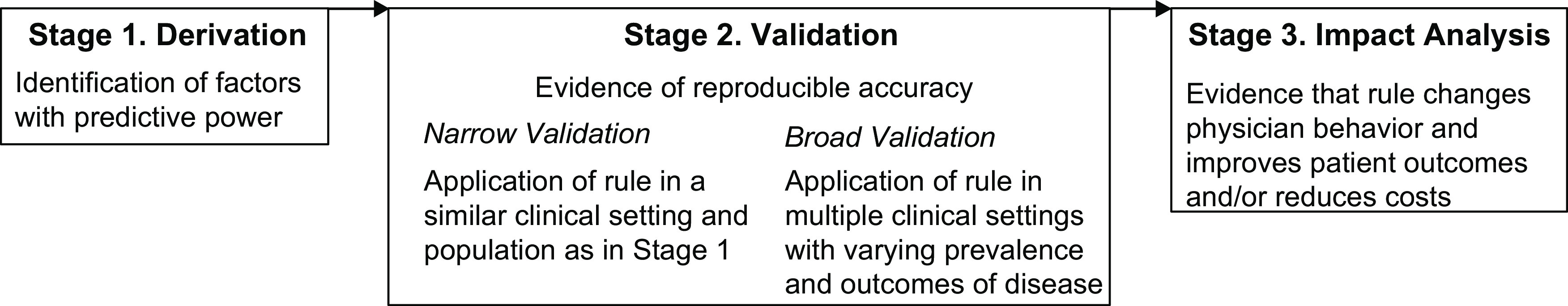
The development process of a clinical decision rule, as conceptualised by McGinn et al (McGinn *et al*., 2000)

Over 20 years of research on the topic of CDRs has led to the formulation of a set of preconditions that must be met by every decision rule. Such a rule should have a clear purpose and be relevant, accurate, concise, and reproducible. Additionally, CDRs should demonstrate content validity; they should be composed of well-recognised, clinically sensible, and independent predictors. Typically, three or more predictor variables should be used, which can be obtained from the patient’s medical history, physical examination, or straightforward diagnostic tests (Graham *et al.,* 2001). These predictors should be incorporated in a rule that is easy to apply and may involve a diagnostic, therapeutic, or preventive course of action, and preferably limits practice variation (le Gal *et al.,* 2012; Rodger *et al.,* 2014). This list of preconditions implies that different stages must be successfully passed when developing a valid and useful CDR. Indeed, at least three stages of CDR development can be distinguished. These three stages are presented in Figure 1, which was initially conceptualised by McGinn et al (2000).

After the primary idea that a simple decision rule may improve decision-making in response to a specific clinical question, the first stage can be initiated. This stage involves the development, or more specifically, the *derivation* of the CDR and its variables, which is subject to strict guidelines (Laupacis *et al.,* 1997; le Gal *et al.,* 2012; McGinn *et al.,* 2000; Rodger *et al.,* 2014; Stiell and Wells, 1999). Typically, this derivation process is supported by a set of specific methodologic standards relating to characteristics such as outcome events, predictors, and study subjects based on original data collection and multivariate statistical analysis. The next step is to confirm the need for the actual newly generated rule after all its details are elaborated, its clinical relevance and ease to use in real life, as well as the expected savings in terms of time and resources (McGinn *et al.,* 2000; Stiell and Wells, 1999). The results of this step may require the rule or the generated score to be refined. An informative illustration of the importance of incorporating this ‘clarification’ step is presented in a publication on the STONE score, a rule whose developers seem to have failed to ask these questions (Green and Schriger, 2016). The authors of the publication question both the need and clinical relevance of the decision rule, stating that it is not clear what its role is in actual clinical decision-making, and that the rule does not outperform clinical judgement.

Once the derivation stage has been completed, a prospective external validation should take place in an independent cohort of patients (le Gal *et al.,* 2012). The rule should be first applied in a narrow setting and in a patient population that is similar to the derivation group in order to validate if the rule’s behaviour is actually the same as in the derivation cohort. Later on, validation in broader, more general, clinical settings is required with varying prevalence and outcomes of disease (McGinn *et al.,* 2000) in order to discover if the rule is suitable for application in these situations. This action should only be done after adequate training of the study physicians, because otherwise, results will most probably be biased by differences in the application of the – hitherto unknown – CDR (Stiell and Wells, 1999). In the end, the total spectrum of patients in which the CDR is to be validated must fit the entire patient – and physician – population in which the CDR is intended to be applied. It is important to ensure that the application of the rule will remain strictly limited to the validated settings and patients, especially after implementation in routine clinical practice (Carmelli *et al.,* 2018) and to confirm that generalisability issues are adequately addressed (Toll *et al.,* 2008).

A CDR should also do what it promises to be valuable in practice. An estimate of the potential impact of use is vital to verify if this is the case (McGinn *et al.,* 2000; Reilly and Evans, 2006; Toll *et al.,* 2008). In such an impact analysis, evidence is collected to demonstrate whether the rule actually changed physicians’ behaviour, improved clinically relevant process parameters or patient outcomes, or reduced costs (le Gal *et al.,* 2012; McGinn *et al.,* 2000). Ideally, an impact study is able to demonstrate a so-called positive resource utilisation impact in an index group, which was exposed to the use of the decision rule, versus a control group, which was subjected to care or clinical judgement as usual. This positive resource utilisation impact amounts to a decrease in the use of the resource in question, for example, diagnostic test ordering, without a corresponding increase in adverse outcomes such as an increase in morbidity/mortality or a decline in quality of life.

Many barriers have been reported between *awareness* of and *adherence* to a new approach (Gaddis *et al.,* 2007). In other words, even if a CDR passed an impact analysis successfully and results have been widely communicated to the professionals involved, this still does not mean that the CDR will actually be implemented in routine daily practice, let alone that it continues to be used in the long run (Laupacis *et al.,* 1997; Reilly and Evans, 2006; Toll *et al.,* 2008; van Doorn and Geersing, 2018).

As a rule of thumb, a CDR will only be embraced by physicians if its use is evidently clinically useful, if the CDR is incorporated into guidelines, if it is, for example, used by both opinion leaders and immediate colleagues, if it results in saving money or time, and if it improves both patients’ and doctors’ lives (Phillips, 2010b). Often, however, not all of these criteria are met. In fact, many factors are known to pose barriers to the use of a CDR. An overview of these factors was selected from the literature^1^ and is presented in Table 1. The Appendix provides an extended version of this table, including some additional context and a few examples.

**Table 1.** Overview of factors that pose barriers for the use of a clinical decision rule in routine clinical practice. NB: the examples that are applicable to a specific CDR differ widely from country to country, as has been demonstrated by the application of the Ottawa Ankle Rules (Graham *et al*., 2001). The Addendum provides an extended version of the table with references, some additional context and a few examples

Barriers to the use of a CDR	Examples
Practical	– too complicated, volatile, rigid, or not accessible enough – no room for clinical reality and clinical context – inability to reconcile patient preferences – too different from current guidelines – another contradictory guideline is on hand – there is an easier alternative for the CDR – external practice constrains apply – regression to the mean
Mental	– interference with professional autonomy^1^ – too much a black box – not convinced of CDR’s reliability or relevance – perceived as impractical – inertia due to previous practice – fear of a trade-off at the expense of patient safety – fear of not being able to meet patient expectations
Financial	– poor reimbursement, increased practice costs – financial incentives for the CDR’s alternative
Legal	– fear of the risk of litigation – regulations conflict with the application of the CDR

1) this might explain why 50% of physicians do not use CDRs at all (Le Marechal *et al*., 2013).

CDR: clinical decision rule.

Due to a combination of these factors, decision rules are poorly implemented in clinical practice; this even applies to the mother of all decision rules, the Ottawa Ankle Rules (Brehaut *et al.,* 2005; Gaddis *et al.,* 2007). Obviously, some factors can be dealt with, for instance, a CDR that is too complicated should be redesigned, while it is to be accepted that others will persist – for instance, the fear of litigation. Most important is to identify which barriers prevent the CDR from being used, and then formulate a targeted strategic approach to overcome these barriers. Many approaches have been suggested (Reilly and Evans, 2006), but most of them have not been proven to be effective. Exceptions thus far include targeted mailing and local implementation strategies equipped with known effective elements, such as audit and feedback, delivered by respected local clinicians (Graham *et al.,* 2001; Stiell and Wells, 1999).

With so many obstacles, it is not surprising that in the end almost all CDRs do not make it to the end^2^. A small subset of CDRs has undergone an appropriate prospective external validation, while only a few have undergone a formal impact analysis (Ban *et al.,* 2021; Ebell *et al.,* 2021; Laupacis *et al.,* 1997; Reilly and Evans, 2006; Toll *et al.,* 2008)^3^. However, once a CDR has passed the entire process, clinicians have an additional tool at their disposal that can be very helpful for them and their patients.

## Decision rules within primary care

Traditionally, CDRs have been implemented primarily in the emergency room, cardiology and paediatrics. Within these clinical settings, in the majority of cases, they serve as a screening tool for safely ruling out a disease outcome prior to imaging (Carmelli *et al.,* 2018; Ebell, 2010; Le Marechal *et al.,* 2013; Lim, 2018; Monahan *et al.,* 2017; Phillips, 2010a; Pugh, 2016; Reed, 2006), next to other clinical tasks of CDRs: diagnosis, prognosis, treatment and prevention (Ebell, 2010).

As previously described, only a few CDRs are regularly used in primary care. An explanation for this phenomenon may be found in the limited number of impact studies that have been performed on the topic of primary care CDRs (Keogh *et al.,* 2014). Decision rules that are known to be routinely applied in primary care in some countries are the Ottawa Ankle Rules and a few cardiovascular rules. Examples of these cardiovascular rules are i) a rule to screen patients with a suspicion of cardiac failure for the need of a cardiac ultrasound (Monahan *et al.,* 2017; Van Riet *et al.,* 2016), ii) the CHA_2_DS_2_-VASc score for determining whether or not treatment is required with anticoagulation therapy or antiplatelet therapy – although this score is mainly used in the hospital (Lucassen, 2018), and iii) the primary care DVT rule, also called the Oudega rule (Oudega *et al.,* 2005b). This rule is widely used in a primary care setting and hence will be discussed in more detail below.

**Figure 2.**
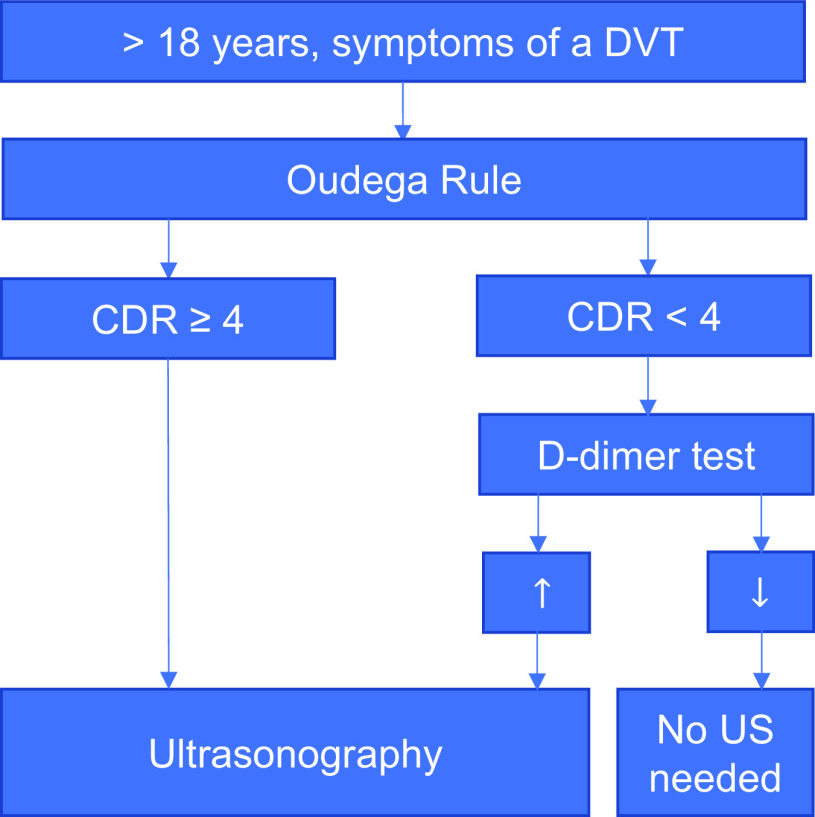
Simplified diagnostic flowchart presenting the clinical application of the primary-care-adapted Wells rule (Oudega rule) for deep vein thrombosis (Oudega *et al*., 2005b). Only a high-risk patient (CDR ≥ 4) is instantly subjected to ultrasonography. In low-risk patients (CDR score < 4), a D-dimer blood test is performed before performing an ultrasound exam. In case of a non-elevated D-dimer test, no ultrasound is needed to safely exclude a DVT. This figure is a simplification of the original flowchart (NHG-werkgroep 2017). DVT: deep vein thrombosis. CDR: clinical decision rule. US: ultrasonography

The Oudega rule was developed for primary care patients and appeared to be better suited for this population than the Wells rule, which was based upon data from hospital outpatients (Oudega *et al.,* 2005b, 2005a; Wells *et al.,* 1997). The rule was developed to rule out DVT, using a clinical score (see Table 2) combined with a D-dimer test (a blood test) to reduce the need for imaging throughout the exclusion process (see Figure 2). In other words, applying the rule ensures that an ultrasound exam is only performed if strictly necessary. Several factors contribute to making this primary care rule a textbook example of a decision rule as intended. These factors will be further explained in the next paragraph.

**Table 2.** Items (risk factors) of the primary-care-adapted Wells rule (Oudega rule) for deep vein thrombosis (Oudega *et al*., 2005b) with their corresponding weight (1 or 2). A total score can be calculated for each patient in order to discriminate low (CDR score < 4) from high (CDR ≥ 4) risk patients. Only a high-risk patient is instantly subjected to ultrasonography. CDR: clinical decision rule

	Risk factors	Weight
1.	Male gender	1
2.	Use of systemic oestrogens (such as contraceptive pills, hormone rings/patches or needles)	1
3.	Presence of malignancy	1
4.	Surgery in the last month	1
5.	Absence of trauma explaining swelling in the calf	1
6.	Expanded veins of the limb	1
7.	Difference in maximum calf size ≥ 3 cm	2

For one, the Oudega rule successfully passed all stages involved in CDR introduction, from derivation to extensive external validation in various settings (Buller *et al.,* 2009; Geersing *et al.,* 2012; Green, 2013; Hendriksen *et al.,* 2015; Oudega *et al.,* 2005b; Toll *et al.,* 2006), as well as several impact analyses (Hendriksen *et al.* 2016; Ten Cate-Hoek *et al.,* 2009; van Maanen *et al.,* 2020). Also, the rule possesses several ‘bonus properties’, making it simpler, more sensible, and more suitable for incorporation in daily practice. These characteristics are explained below.

- The first feature is the fact that the Oudega rule is both a dichotomised and a two-way rule. The dichotomisation involves a classification into only two groups: a low-risk and a high-risk group (Reilly and Evans, 2006). In a two-way rule, a recommendation is made for each of the patient groups, whereas in a one-way rule, a patient either meets the criteria and the CDR recommends a specific outcome or the patient does not meet the criteria, and no recommendation is made. This can be confusing, leading to inability to act and can, paradoxically, result in an increase in testing (Carmelli *et al.,* 2018; Green, 2013). In the Oudega rule, a recommendation describing a clear course of action – ordering a D-dimer test versus a radiologic exam – is made for both patient groups (low-risk and high-risk), which makes it a two-way rule.
- The second advantage is the fact that the rule was designed for exclusion (rule-out) rather than for inclusion (detection). Clinicians are generally much more comfortable with a rule that is primarily able to correctly rule out an illness (high sensitivity^4^) than with a rule that is designed to efficiently detect an illness (high specificity^5^) (Ebell, 2010; Laupacis *et al.,* 1997; Reilly and Evans, 2006). This preference can be explicated by the fact that clinicians are more eager not to miss a diagnosis than about making the diagnostic process more efficient. Subsequently, the benefit of using the rule itself lies in a specificity that measurably outperforms clinical judgement with a comparable high sensitivity (Carmelli *et al*., 2018; Green and Schriger, 2016), as is indeed the case of the Oudega rule (Geersing *et al*., 2010; van Maanen *et al*., 2020).
- Lastly, there is a substantial difference between the risk of disease of patients classified as low risk versus those classified as high risk. Such a difference makes it worthwhile to actually apply the rule (Ebell *et al.,* 2021). The probability of disease in the low-risk-DVT group followed by a negative D-dimer test, ≈1.5%^6^, is low enough to require only a D-dimer test for these primary care patients and taking the (small) risk of a false-negative result for granted (Kingma *et al.,* 2017). Simultaneously, in the high-risk group, performing an instant ultrasound exam, which is more accurate but also more expensive and time-consuming than a D-dimer test, can be justified by an actual risk, ≈33%, of suffering from a clinically relevant thrombo-embolic event.

Once more, it appeared that applying a CDR in daily practice has limitations that are not addressed in study settings. In practice, it is found that the Oudega rule is often used incorrectly (Kingma *et al.,* 2017; van Maanen *et al.,* 2020), or it is used in only a limited number of eligible patients (Kingma *et al.,* 2017). These factors contribute to a lower efficiency and higher failure rate of the diagnostic work-up (Geersing *et al.,* 2010; van Maanen *et al.,* 2020) than in the case of optimal use of the CDR.

This limited adoption of the rule may occur due to several causes. The most obvious cause for the suboptimal compliance with this primary care rule seems to be the time pressure in the primary care setting. During busy office hours in a crowded practice, GPs are tempted to base estimates on experience and clinical judgments, or only to use those clinical parameters that are readily available (Seaberg, 2001; Toll *et al.,* 2008; Xu *et al.,* 2021). Moreover, this effect is enhanced by a low exposure rate of DVT suspicions, combined with a rule that incorporates a relatively high number of items (Green, 2013), seven of which at least one is considered non-trivial by many GPs^7^ (le Gal *et al.,* 2012). Based on these items (Table 2), in case of a low clinical score, the diagnostic procedure requires a D-dimer test prior to further diagnostic testing, and the need to refer to a laboratory can be an obstacle when an urgent decision has to be made.

On closer inspection, however, these issues do not seem insurmountable.

First, both the trigger of the application of the CDR and the memorisation of its content can be taken over by computer software or personnel other than the primary care physician. Such can be realised by the introduction of a DVT care pathway (DCP), in which an elevated D-dimer test, carried out at the phlebotomy service, is directly followed by an ultrasound exam without the intervention of the GP (Heerink *et al.,* 2022). In the DCP electronic request form, a window pops-up containing the CDR and its items, which need not necessarily be filled out by the physician that orders this protocol. Besides, it has been demonstrated that the mere application of a CDR in patients with suspected DVT, in the context of a DVT care pathway, acts as a ‘selection filter’, potentially preventing many unnecessary D-dimer tests from being performed (Heerink *et al.,* 2022).

Also, applying the latest medical insights to the Oudega criteria may lead to a modification (update) of this CDR, which is essentially untouched for about 25 years while it is known that CDRs in general can become ‘out of date’ rapidly due to new insights or new tests (le Gal *et al.,* 2012; Toll *et al.,* 2008). Indeed, a recent study proposed a simplification of the current rule consisting of a D-dimer test and two simple items that can be applied even more rapidly in primary care (Xu *et al.,* 2021), similar to the YEARS score for hospital outpatients with a suspicion of pulmonary embolism (van der Hulle *et al.,* 2017; Van Es *et al.,* 2015). Even the application of D-dimer as a stand-alone test –without a CDR– for all patients with suspected DVT seems to be safe and efficient, as recent data have been demonstrated (Rinde *et al.,* 2020)^8^.

Lastly, promising new diagnostic opportunities are emerging, such as easy-to-use point-of-care (POC) D-dimer tests, which enable performing a D-dimer test, preceded or not by a CDR, in a one-stop visit at the GP’s office (Ellis *et al.,* 2021; Heerink *et al.,* 2020; Price *et al.,* 2021). In this way, the time delay that comes with referring the patient to the laboratory for a D-dimer test can be eliminated, potentially lowering the threshold for using a CDR.

Taking all these trends into consideration, it is difficult to say which arguments will ultimately determine whether or not a rule like in this case the Oudega rule will continue to exist in its present form.

Generally speaking, one can postulate that the opposite is also true: changing conditions may lead to new opportunities for introducing sensible CDRs, or for making current CDRs more useful. For example, due to the SARS-CoV-2 pandemic, visits to the GP’s office have been replaced increasingly by time-consuming home visits. The time spent on these home visits may be decreased by using CDRs that can be used remotely and, if necessary, be combined with a POC test on the spot. Accordingly, the added value of such CDRs will be immediately visible, since patients could be discharged instantaneously in case of a favourable CDR score. As a result, such a practical benefit will most probably increase compliance among physicians, which is in turn a key success factor in a fruitful implementation process of a CDR. Hence, by taking advance of such new trends in the development of future decision rules, primary care CDRs could still be incentivised to maximally exploit the simple nature of CDRs.

## Conclusion

In conclusion, the potential benefits of using CDRs in primary care are numerous but only come into their own if tailor-made CDRs are being developed and used that take the specific context of primary care into consideration, and if they anticipate the latest developments such as the use of POC tests. Current relevancy of the few CDRs in use should be evaluated periodically. Accordingly, new powerful well-validated primary care CDRs can make a meaningful contribution to improving the quality of primary health care and patient satisfaction while reducing unnecessary costs.

## Footnotes

1 Selection of literature was based on publications obtained through a PubMED search performed in October 2022 using the entry term ‘Clinical Decision Rules’, including its MeSH terms. A final selection of relevant publications was based on titles and abstracts, successively.

2 Almost 20 years ago, estimates of scientific articles discussing CDRs already exceeded 15 000 (Toll *et al.,* 2008). Nowadays, numbers have probably been increased to a multiple of that amount.

3 A recent study has demonstrated that in cardiology, a field in which traditionally many CDRs are being used, only 110 impact studies have been performed, from which a small minority has been described as ‘studies of sufficient quality’ (Ban *et al.,* 2021).

4 sensitivity refers to the proportion of patients having the outcome of interest in whom the results are abnormal

5 specificity refers to the proportion of patients who do not have the outcome of interest in whom the results are normal

6 prevalence of disease in the low-risk group as a whole is ≈10%, but this percentage is less relevant since the D-dimer is nowadays an essential element of the work-up in this patient category

7 an example of such an item is the estimation of the calf size (see table 2)

8 As a side note, it should be mentioned that findings of the studies that propagate a radical simplification of the current decision rule (Rinde *et al.,* 2020; Xu *et al.,* 2021) need to be confirmed in prospective management studies, especially in primary care settings. In fact, an over-simplified decision rule tends to decrease in sensitivity.

## Appendix

**Table:**

Category	Examples
Practical	– CDR is too complicated, volatile, rigid, or not accessible enough, or it is too tempting to interpret the CDR otherwise than intended (Brehaut *et al.,* 2005; Cabana *et al.,* 1999) – clinical reality and clinical context cannot be adequately expressed in the CDR (van Doorn and Geersing, 2018) – inability to reconcile patient preferences with the CDR (i.e., patients may be resistant or perceive no need for it, or perceive the CDR as offensive or embarrassing) (Brehaut *et al.,* 2005; Cabana *et al,.* 1999) – CDR is too different from current guidelines, or it is not possible to try it out or to observe its use from immediate colleagues (Stiell and Wells, 1999) – another contradictory guideline is on hand (Cabana *et al.,* 1999) – there is an easier alternative for the CDR (i.e., it is more convenient to simply order a radiograph or to treat a patient instead of going through the time-consuming process of applying a CDR) (Abboud and Cabana, 2001; Seaberg, 2001) – external practice constrains apply for the CDR (i.e., time limitations, lack of a counselling materials or a reminder system, insufficient staff or consultant support) (Abboud and Cabana, 2001; Cabana *et al.,* 1999) – limited added value of CDR due to ‘regression to the mean’ after long-term use of the CDR (Reilly and Evans, 2006)
Mental	– ‘intuitive’ gestalt appeals to professional autonomy, whereas ‘rational’ cookbook medicine does not (Brehaut *et al*., 2005; Cabana *et al*., 1999; Graham *et al*., 2001; Phillips, 2010b; Reilly and Evans, 2006)^1^ – CDR is too much a black box to be accepted (Ebell, 2010) – clinicians are not convinced that the CDR either successfully passed earlier research stages (e.g., impact study), or that it is applicable to the clinicians’ patient population, they ignore population-level advantages, do not see their own decisions as the root cause of the problem or they do not consider the underlying issue a problem at all (Cabana *et al.,* 1999; Phillips, 2010b; Reilly and Evans, 2006) – inertia due to previous practice (e.g., habits, routines) (Abboud and Cabana, 2001) – fear of a trade-off at the expense of patient safety or other unanticipated consequences when using the CDR, especially if the rule recommends elimination of an established behaviour (Cabana *et al.,* 1999; Reilly and Evans, 2006) – clinicians assume that patients expect a radiograph to be ordered instead of a time-consuming discussion about the need for one (Gaddis *et al.,* 2007)
Financial	– poor reimbursement, increased practice costs (Cabana *et al.,* 1999) – financial incentives for the CDR’s alternative (e.g., for starting therapy) (Ebell, 2010; Stiell and Wells, 1999)
Legal	– fear of the risk of litigation when applying the CDR (e.g., if this implies a patient is denied a radiograph or therapy) (Abboud and Cabana, 2001; Ebell, 2010; McGinn *et al.,* 2000; Stiell and Wells, 1999) – regulations conflict with the application of the CDR

Factors that pose barriers for the use of a clinical decision rule in routine clinical practice. NB: the examples that are applicable to a specific CDR differ widely from country to country, as has been demonstrated by the application of the Ottawa Ankle Rules (Graham *et al*., 2001).

1) this might explain why 50% of physicians do not use CDRs at all (Le Marechal *et al*., 2013).

CDR: clinical decision rule.

## References

[ref1] Abboud PA and Cabana MD (2001) Understanding barriers to the adoption of clinical decision rules. Annals of Emergency Medicine 38, 703–704. 11719755 10.1067/mem.2001.119945

[ref2] Ban J-W , Chan MS , Muthee TB , Paez A , Stevens R and Perera R (2021) Design, methods, and reporting of impact studies of cardiovascular clinical prediction rules are suboptimal: a systematic review. Journal of Clinical Epidemiology 133, 111–120. 33515655 10.1016/j.jclinepi.2021.01.016

[ref3] Brehaut JC , Stiell IG , Visentin L and Graham ID (2005) Clinical decision rules “in the real world”: how a widely disseminated rule is used in everyday practice. Academic Emergency Medicine 12, 948–956. 16166599 10.1197/j.aem.2005.04.024

[ref4] Buller HR , Ten Cate-Hoek AJ , Hoes AW , Joore MA , Moons KGM , Oudega R , Prins MH , Stoffers HEJH , Toll DB , van der Velde EF and van Weert HCPM (2009) Safely ruling out deep venous thrombosis in primary care. Annals of Internal Medicine 150, 229–235. 19221374

[ref5] Cabana MD , Rand CS , Powe NR , Wu AW , Wilson MH , Abboud PA and Rubin HR (1999) Why don’t physicians follow clinical practice guidelines?: A framework for improvement. Jama 282, 1458–1465. 10535437 10.1001/jama.282.15.1458

[ref6] Carmelli G , Grock A , Picart E and Mason J (2018) The nitty-gritty of clinical decision rules. Annals of Emergency Medicine 71, 711–713. 29776497 10.1016/j.annemergmed.2018.04.004

[ref7] Ebell M (2010) AHRQ White Paper: use of clinical decision rules for point-of-care decision support. Medical Decision Making 30, 712–721. 21183758 10.1177/0272989X10386232

[ref8] Ebell MH , Rahmatullah I , Cai X , Bentivegna M , Hulme C , Thompson M and Lutz B (2021) A systematic review of clinical prediction rules for the diagnosis of influenza. The Journal of the American Board of Family Medicine 34, 1123–1140. 34772768 10.3122/jabfm.2021.06.210110

[ref9] Ellis JE , Johnston TW , Craig D , Scribner A , Simon W and Kirstein J (2021) Performance evaluation of the Quantitative Point-of-Care LumiraDx D-Dimer Test. Cardiology and Therapy 10, 547–559. doi: 10.1007/s40119-021-00241-7. 34618321 PMC8496146

[ref10] Gaddis GM , Greenwald P and Huckson S (2007) Toward improved implementation of evidence-based clinical algorithms: clinical practice guidelines, clinical decision rules, and clinical pathways. Academic Emergency Medicine 14, 1015–1022. 17967964 10.1197/j.aem.2007.07.010

[ref11] Geersing GJ , Erkens PM , Lucassen WAM , Büller HR , Ten Cate H , Hoes AW , Moons KGM , Prins MH , Oudega R , van Weert HCPM and Stoffers HEJH (2012) Safe exclusion of pulmonary embolism using the Wells rule and qualitative D-dimer testing in primary care: prospective cohort study. Bmj 345, e6564. 10.1136/bmj.e6564 PMC3464185 23036917

[ref12] Geersing GJ , Janssen KJ , Oudega R , van Weert H , Stoffers H , Hoes A and Moons K (2010) Diagnostic classification in patients with suspected deep venous thrombosis: physicians’ judgement or a decision rule? British Journal of General Practice 60, 742–748. 10.3399/bjgp10X532387 PMC2944933 20883623

[ref13] Graham ID , Stiell IG , Laupacis A , McAuley L , Howell M , Clancy M , Durieux P , Simon N , Emparanza JI , Aginaga JR , O’connor A and Wells G (2001) Awareness and use of the Ottawa ankle and knee rules in 5 countries: can publication alone be enough to change practice? Annals of Emergency Medicine 37, 259–266. 11223761 10.1067/mem.2001.113506

[ref14] Green SM (2013) When do clinical decision rules improve patient care? Annals of Emergency Medicine 62, 132–135. 23548403 10.1016/j.annemergmed.2013.02.006

[ref15] Green SM and Schriger DL (2016) The sinking STONE: what a failed validation can teach us about clinical decision rules. Annals of Emergency Medicine 67, 433–436. 26803703 10.1016/j.annemergmed.2015.11.022

[ref16] Heerink JS , Gemen E , Oudega R , Hopstaken R , Geersing GJ and Kusters R (2020) Analytical performance and user-friendliness of five novel point-of-care D-dimer assays. Scandinavian Journal of Clinical and Laboratory Investigation 27, 1–8. 10.1080/00365513.2020.1768586 32459511

[ref17] Heerink JS , Péquériaux N , Oudega R , de Jong M , Koffijberg H and Kusters R (2022) Implementation of a care pathway for deep vein thrombosis: what’s the benefit? Thrombosis Update, 100109.

[ref18] Hendriksen JM , Geersing GJ , Van Voorthuizen SC , Oudega R , Ten Cate-Hoek AJ , Joore MA , Moons KGM and Koffijberg H (2015) The cost-effectiveness of point-of-care D-dimer tests compared with a laboratory test to rule out deep venous thrombosis in primary care. Expert Review of Molecular Diagnostics 15, 125–136. doi: 10.1586/14737159.2015.976202. 25537569

[ref19] Hendriksen JMT , Lucassen WAM , Erkens PMG , Stoffers HEJH , van Weert HCPM , Büller HR , Hoes AW , Moons KGM and Geersing GJ (2016) Ruling out pulmonary embolism in primary care: comparison of the diagnostic performance of “gestalt” and the wells rule. Annals of Family Medicine 14, 227–234. doi: 10.1370/afm.1930. 27184993 PMC4868561

[ref20] Keogh C , Wallace E , O’Brien KK , Galvin R , Smith SM , Lewis C , Cummins A , Cousins G , Dimitrov BD and Fahey T (2014) Developing an international register of clinical prediction rules for use in primary care: a descriptive analysis. The Annals of Family Medicine 12, 359–366. 25024245 10.1370/afm.1640 PMC4096474

[ref21] Kingma AEC , van Stel HF , Oudega R , Moons KGM and Geersing GJ (2017) Multi-faceted implementation strategy to increase use of a clinical guideline for the diagnosis of deep venous thrombosis in primary care. Family Practice 34, 446–451. 27471223 10.1093/fampra/cmw066

[ref22] Laupacis A , Sekar N and Stiell IG (1997) Clinical prediction rules: a review and suggested modifications of methodological standards. Jama 277, 488–494. 9020274

[ref23] le Gal G , Carrier M and Rodger M (2012) Clinical decision rules in venous thromboembolism. Best Practice \& Research Clinical Haematology 25, 303–317. 22959547 10.1016/j.beha.2012.06.001

[ref24] Le Marechal F , Martinot A , Duhamel A , Pruvost I and Dubos F (2013) Streptococcal pharyngitis in children: a meta-analysis of clinical decision rules and their clinical variables. BMJ Open 3, e001482. 10.1136/bmjopen-2012-001482 PMC3612811 23474784

[ref25] Lim SH (2018) Clinical decision rules in emergency care. Singapore Medical Journal 59, 169. 29749424 10.11622/smedj.2018042 PMC5915630

[ref26] Lucassen W (2018) CHA2DS2-VASc-risicoscore geen statisch gegeven. *Huisarts En Wetenschap*.

[ref27] McGinn TG , Guyatt GH , Wyer PC , Naylor CD , Stiell IG and Richardson WS (2000) Users’ guides to the medical literature: XXII: how to use articles about clinical decision rules. Jama 284, 79–84. 10872017 10.1001/jama.284.1.79

[ref28] Monahan M , Barton P , Taylor CJ , Roalfe AK , Richard Hobbs FD , Cowie M , Davis R , Deeks J , Mant J , McCahon D , McDonagh T , Sutton G and Tait L (2017) MICE or NICE? An economic evaluation of clinical decision rules in the diagnosis of heart failure in primary care. International Journal of Cardiology 241, 255–261. 28366472 10.1016/j.ijcard.2017.02.149 PMC5483229

[ref29] NHG-werkgroep (2017) NHG-Standaard Diepe veneuze trombose en longembolie. *Huisarts Wet 2017*, 60.

[ref30] Oudega R , Hoes AW and Moons KG (2005a) The Wells rule does not adequately rule out deep venous thrombosis in primary care patients. Annals of Internal Medicine 143, 100–107. 16027451 10.7326/0003-4819-143-2-200507190-00008

[ref31] Oudega R , Moons KG and Hoes AW (2005b) Ruling out deep venous thrombosis in primary care. A simple diagnostic algorithm including D-dimer testing. Thrombosis and Haemostasis 94, 200–205. 16113804 10.1160/TH04-12-0829

[ref32] Phillips B (2010a) Clinical decision rules: how to build them. Archives of Disease in Childhood-Education and Practice 95, 83–87. 10.1136/adc.2009.174441 20501531

[ref33] Phillips B (2010b) Clinical decision rules: how to use them. Archives of Disease in Childhood-Education and Practice 95, 88–92. 10.1136/adc.2009.174458 20501532

[ref34] Price CP , Fay M and Hopstaken RM (2021) Point-of-Care Testing for D-Dimer in the Diagnosis of Venous Thromboembolism in Primary Care: A Narrative Review. Cardiology and Therapy 10, 27–40. doi: 10.1007/S40119-020-00206-2. 33263839 PMC8126530

[ref35] Pugh MJ (2016) Clinical decision rules for epilepsy care: The case for thinking big. *Epilepsy \& Behavior*, Elsevier, 220–221. 10.1016/j.yebeh.2016.02.034 26979764

[ref36] Reed MH (2006) Clinical decision rules in radiology. Academic Radiology 13, 562–565. 16627196 10.1016/j.acra.2006.01.053

[ref37] Reilly BM and Evans AT (2006) Translating clinical research into clinical practice: impact of using prediction rules to make decisions. Annals of Internal Medicine 144, 201–209. 16461965 10.7326/0003-4819-144-3-200602070-00009

[ref38] Rinde FB , Fronas SG , Ghanima W , Vik A , Hansen J-B and Brækkan SK (2020) D-dimer as a stand-alone test to rule out deep vein thrombosis. Thrombosis Research 191, 134–139. 32447095 10.1016/j.thromres.2020.04.026

[ref39] Rodger MA , Le Gal G , Wells P , Baglin T , Aujesky D , Righini M , Palareti G , Huisman M and Meyer G (2014) Clinical decision rules and D-Dimer in venous thromboembolism: current controversies and future research priorities. Thrombosis Research 134, 763–768. 25129416 10.1016/j.thromres.2014.07.031

[ref40] Seaberg DC (2001) Truth and clinical decision rules. Academic Emergency Medicine 8, 190–191. 11157299 10.1111/j.1553-2712.2001.tb01288.x

[ref41] Stiell IG and Wells GA (1999) Methodologic standards for the development of clinical decision rules in emergency medicine. Annals of Emergency Medicine 33, 437–447. 10092723 10.1016/s0196-0644(99)70309-4

[ref42] Ten Cate-Hoek AJ , Toll DB , Büller HR , Hoes AW , Moons KGM , Oudega R , Stoffers HEJH , van der Velde EF , van Weert HCPM , Prins MH and Joore MA (2009) Cost-effectiveness of ruling out deep venous thrombosis in primary care versus care as usual. Journal of Thrombosis and Haemostasis 7, 2042–2049. 19793189 10.1111/j.1538-7836.2009.03627.x

[ref43] Toll DB , Janssen KJM , Vergouwe Y and Moons KGM (2008) Validation, updating and impact of clinical prediction rules: a review. Journal of Clinical Epidemiology 61, 1085–1094. 19208371 10.1016/j.jclinepi.2008.04.008

[ref44] Toll DB , Oudega R , Bulten RJ , Hoes AW and Moons KG (2006) Excluding deep vein thrombosis safely in primary care. Journal of Family Practice 55, 613–618. 16822449

[ref45] van der Hulle T , Cheung WY , Kooij S , Beenen LFM , van Bemmel T , van Es J , Faber LM , Hazelaar GM , Heringhaus C , Hofstee H , Hovens MMC , Kaasjager KAH , van Klink RCJ , Kruip MJHA , Loeffen RF , Mairuhu ATA , Middeldorp S , Nijkeuter M , van der Pol LM , Schol-Gelok S , Ten Wolde M , Klok FA and Huisman MV (2017) Simplified diagnostic management of suspected pulmonary embolism (the YEARS study): a prospective, multicentre, cohort study. Lancet 390, 289–297. 28549662 10.1016/S0140-6736(17)30885-1

[ref46] van Doorn S and Geersing G-J (2018) De huisarts, atriumfibrilleren en falende beslisregels. Huisarts En Wetenschap 61, 27–29.

[ref47] Van Es J , Beenen LFM , Douma RA , den Exter PL , Mos ICM , Kaasjager HAH , Huisman MV , Kamphuisen PW , Middeldorp S and Bossuyt PMM (2015) A simple decision rule including D-dimer to reduce the need for computed tomography scanning in patients with suspected pulmonary embolism. Journal of Thrombosis and Haemostasis 13, 1428–1435. 25990714 10.1111/jth.13011

[ref48] van Maanen R , Kingma AEC , Oudega R , Rutten FH , Moons K and Geersing G-J (2020) Real-life impact of clinical prediction rules for venous thromboembolism in primary care: a cross-sectional cohort study. BMJ Open 10, e039913. 10.1136/bmjopen-2020-039913 PMC7772307 33372074

[ref49] Van Riet EES , Hoes AW , Limburg A , Landman MAJ , Kemperman H and Rutten FH (2016) Extended prediction rule to optimise early detection of heart failure in older persons with non-acute shortness of breath: a cross-sectional study. BMJ Open 6, e008225. 10.1136/bmjopen-2015-008225 PMC4762114 26880668

[ref50] Wells PS , Anderson DR , Bormanis J , Guy F , Mitchell M , Gray L , Clement C , Robinson KS and Lewandowski B (1997) Value of assessment of pretest probability of deep-vein thrombosis in clinical management. The Lancet 350, 1795–1798. 10.1016/S0140-6736(97)08140-3 9428249

[ref51] Xu K , de Wit K , Geersing G-J , Takada T , Schutgens R , Elf J , Kearon C and Parpia S (2021) A simplified decision rule to rule out deep vein thrombosis using clinical assessment and D-dimer. Journal of Thrombosis and Haemostasis 19, 1752–1758. 33834620 10.1111/jth.15337

